# Excess Cardiovascular Risk in Diabetic Women: A Case for Intensive Treatment

**DOI:** 10.1007/s11906-015-0554-0

**Published:** 2015-04-24

**Authors:** C. Recarti, S. J. S. Sep, C. D. A. Stehouwer, T. Unger

**Affiliations:** CARIM—School for Cardiovascular Diseases, Maastricht University, Maastricht, The Netherlands

**Keywords:** Diabetes, Cardiovascular risk, Women, Sex differences

## Abstract

Diabetes is a common and rapidly growing disease that affects more than 380 million people worldwide and is an established risk factor for cardiovascular disease with differential effects on women compared to men. While the general population of women, particularly young women, has more favourable cardiovascular risk profiles than men, this protective effect has been shown to be lost or even reversed in diabetic women. Several studies have demonstrated a significant diabetes-associated excess risk of cardiovascular disease in women. Sex-specific differences in risk factors associated with diabetes and their management may be responsible for the relative excess cardiovascular risk in women with diabetes. Diabetic women need intensive treatment in order to optimize management of cardiovascular risk factors. Further studies are needed to elucidate the mechanisms underlying the excess cardiovascular risk in diabetic women in order to tailor prevention and treatment strategies.

## Introduction

Diabetes is a common and rapidly growing disease [1]. The International Diabetes Federation has projected that the number of people with diabetes in the world will increase from 387 million in 2014 to 592 million by 2035 [2]. The World Health Organization (WHO) reported a global prevalence of 9 % in 2014 [3]. There are two main forms of diabetes. Type 1 diabetes is due primarily to auto-immune-mediated destruction of pancreatic beta-cells resulting in insulin deficiency. Type 2 diabetes is characterized by insulin resistance and abnormal insulin secretion and accounts for 85–95 % of diabetes cases globally [4•]. Both types of diabetes are on the increase, type 2 diabetes in particular.

Apart from the heightened genetic susceptibility of certain ethnic groups, environmental and behavioural factors such as sedentary lifestyle and nutrition are responsible for the diabetes epidemic.

Individuals with diabetes have an increased risk of developing a number of disabling and life-threatening health problems, including serious diseases affecting the cardiovascular system, eyes, kidneys, and nerves. Evidence for an excess risk of cardiovascular disease associated with diabetes in women is increasing, an observation that urges intensified treatment and management of female diabetes patients. This review summarizes the evidence of sex differences in diabetes-associated risk of cardiovascular disease and provides possible explanations for the excess risk in women that may provide female-specific targets for prevention and treatment. It also highlights the importance of sex-specific analysis in trials and observational studies of treatment and control of cardiovascular risk factors in persons with diabetes and gives recommendations for future research.

## Epidemiology of Diabetes

Type 1 diabetes occurs predominantly in children and young adults. Incidence rates vary widely and range from 0.1/100,000 per year in Chinese regions to more than 40/100,000 per year in Finland [5]. Age-specific incidence rates do not differ between the sexes up to 14 years of age in most studies [6]. However, at older ages, male excess is a constant finding in populations of European origin [7–10].

Type 2 diabetes is a multifactorial and heterogeneous disease. It is often a manifestation of clustering of disorders, including hyperinsulinaemia, dyslipidaemia, hypertension, visceral obesity, hypercoagulability and microalbuminuria. The diabetes epidemic and the potential for increases in numbers of cases, although apparent around the world, are most pronounced in non-Caucasian populations [11]. The global prevalence of diabetes was 8 % in 2013 and reached 35 % in some regions [4]. Overall, there is little sex difference in the global numbers of people with type 2 diabetes. The prevalence rises with age and is slightly higher in men up to the age of 70 [4]. The global prevalence in men 50 years of age was about 15 % compared to 13 % in their female counterparts. Due to the long time period that frequently elapses before diabetes is detected, a substantial number of cases may be undiagnosed.

Diabetes is a major global cause of premature death that is easily underestimated because only a minority of patients with diabetes die from a cause uniquely related to the condition. Diabetes is a stronger risk factor for all-cause mortality in women than in men [12]. According to global WHO estimates, in 2012, diabetes caused 684,346 deaths in men and 813,025 deaths in women, representing 2.3 and 3.1 % of all deaths in men and women, respectively [3]. However, the number of deaths caused by diabetes could be underestimated because attributing deaths to diabetes is challenging. Using a modelling approach, the International Diabetes Federation estimated that the number of deaths caused by diabetes in 2013 was 5.1 million among people aged 20–79 years, accounting for 8.4 % of global all-cause mortality among people in this age group [4, 13]. Diabetes accounts for a larger proportion of deaths in women than in men over 49 years of age, affecting over 25 % of women in some regions and age groups [13].

## Excess Cardiovascular Risk in Women with Diabetes

The leading cause of morbidity and mortality among people with diabetes is cardiovascular disease [14, 15]. Approximately one half of individuals with type 2 diabetes will die of a cardiovascular cause [16]. It has been reported that diabetes confers an equivalent risk to ageing 15 years [17]. In the general population, cardiovascular disease events occur years later in women than in men. In 2012, a large study evaluating the impact of sex on cardiovascular outcomes in high-risk patients under optimal standard treatment (*n* = 31,546; 30 % women; >55 years) demonstrated better outcomes in women than in men [18••]. Kappert et al. combined two parallel trials (ONTARGET and TRANSCEND) cohorts of individuals at high risk of a major cardiovascular event on the basis of one or more of the following: history of coronary artery disease, peripheral vascular disease, stroke or transient ischemic attack, or diabetes mellitus with end-organ complications. The combined risk of cardiovascular death, myocardial infarction (MI) and stroke was significantly lower (−21 %) in women than in men. Similarly, the risk for MI was 22 % lower in female than in male patients. Furthermore, women experienced the end points on average 5 to 10 years later [18••]. This finding is in line with data obtained from the INTERHEART study (*n* = 27,098; 25 % women), a case-control study that showed a delay of 9 years in occurrence of the first acute MI in women compared to men [19].

Studies have consistently found that individuals with diabetes have a higher risk of morbidity and mortality due to cardiovascular disease compared with those without diabetes [14, 15]. Moreover, several studies have demonstrated that diabetes greatly attenuates, or may even reverse, the protective effect of female sex on cardiovascular risk [17, 18••, 19]. The study of Kappert et al. [18••], in agreement with the INTERHEART study [19], showed that diabetes altered the sex differences in cardiovascular outcomes: while the risk of MI was substantially lower in female vs. male non-diabetic persons, diabetic women were at significantly greater risk of developing MI compared to diabetic men. In a recent meta-analysis, the excess risk of fatal cardiovascular disease in persons with type 1 diabetes was twice as high in women compared with men [20••]. In the past 15 years, three meta-analyses have examined the relative risk of fatal coronary heart disease associated with diabetes in women vs. men. All these studies point toward an increased risk in women, but only two of the three reported a significant difference (up to 50 %) after adjustment for classic cardiovascular risk factors [21–23]. In 2014, a meta-analysis (data from 64 studies, *n* = 858,507 individuals, *n* = 28,203 events) showed that the relative risk of incident coronary heart disease was 44 % greater in diabetic women than in diabetic men [24••]. Another meta-analysis (data from 7 studies, *n* = 59,383 individuals, *n* = 1427 events) found an even greater (2.5-fold) excess risk of incident coronary heart disease in women compared to men with type 1 diabetes [20••].

Stroke is another well-known diabetes-associated outcome [25]. In contrast to MI, contradictory findings have been reported on sex differences in diabetes-related risk for stroke, claiming decreased [26, 27], equal [28] or increased [29, 30] risk in diabetic women compared with men. However, the most recent systematic reviews reported that the excess risk of stroke is 27 % greater in women with diabetes of all types (data from 64 studies, *n* = 775,385 individuals, *n* = 12,539 events) [31] and 37 % greater in women with type 1 diabetes (data from 4 studies, *n* = 45,677 individuals, *n* = 371 events) [20••] compared with their male counterparts, independent of sex differences in other major cardiovascular risk factors. Diabetes has also been found to increase cardiovascular events and death after stroke [32–34]. A study based on the Northern Sweden MONICA Stroke Registry showed reduced survival after stroke in diabetic patients compared to non-diabetic patients and an even more pronounced reduction in survival in diabetic women [35]*.*

The mechanisms responsible for these sex differences in diabetes-related cardiovascular risk remain to be elucidated.

## Possible Causes of the Sex Differences in Diabetes-Associated Cardiovascular Risk

Multiple mechanisms may be responsible for the sex difference in diabetes-associated risk of cardiovascular disease. Postulated underlying causes include increases in the prevalence of classical cardiovascular risk factors and in the effects of the risk factors on the development of cardiovascular disease in diabetic persons, decreases in risk factor control, and interference with protective mechanisms in the vascular wall in women with diabetes.

### Cardiovascular Risk Factors

It has been postulated that women have to ‘travel further’ than men to develop diabetes [24••]. Observed differences in prevalence of risk factors in individuals with diabetes compared with those without diabetes are often greater in women. These risk factors include waist-to-hip ratio, obesity, insulin resistance, dyslipidaemia, hypertension, coagulation factors, endothelial dysfunction, and systemic inflammation [36–39]. Some cardiovascular risk factors may have a greater effect on absolute risk in diabetic women than in diabetic men [38].

Endothelial function has been shown to be impaired to a greater extent in premenopausal women with type 2 diabetes than in men with type 2 diabetes [40]. Furthermore, low-grade inflammation may have a greater role in perturbing insulin action in women, and inflammatory factors may interact with female sex hormones, resulting in a decrease in the protective effects of oestrogens on body fat distribution and insulin action [41].

### Management, Treatment and Control of Cardiovascular Risk Factors

In persons with diabetes, a gender disparity in the treatment and control of cardiovascular risk factors has been reported [24••, 42, 43]. However, these treatment differences are less evident in more recent studies, and some evidence of higher treatment rates in women has emerged [18••, 44–46•]. Even when treated similarly, control of hyperglycaemia and major cardiovascular risk factors appears to be less satisfactory in women than in men. Data from medical records of 286,791 Spanish patients with type 2 diabetes (153,987 men and 132,804 women, of whom 22.3 and 13.8 % had prior cardiovascular disease, respectively), control of HbA1c, blood pressure and LDL cholesterol was worse in women, with or without prior cardiovascular disease, although differences were small [44]. In a cross-sectional survey at 10 outpatient diabetes clinics in Italy (MIND.IT Study, *n* = 1297 men and *n* = 1168 women with no previous cardiovascular events), optimal targets for HbA1c, LDL cholesterol, HDL cholesterol and systolic blood pressure were significantly less frequently achieved by women than by men after adjustment for overweight/obesity and age [45]. Similarly, an Italian study of patients with type 2 diabetes attending 19 hospital-based diabetes clinics (RIACE Study, *n* = 15,773) revealed less successful achievement of treatment targets for HbA1c, LDL, HDL and non-HDL cholesterol, and obesity in women than in men, even after adjustment for treatment, BMI, duration of diabetes and age [46•]. The difference in systolic blood pressure control was not significant after adjustment for age.

The observation that women with diabetes are less frequently on target for cardiovascular risk factors suggests that the increased treatment rate in women is insufficient to bridge the gap in cardiovascular risk burden between the sexes. These findings and the lack of evidence for reduced drug efficacy in women compared with men suggest the involvement of other mechanisms. Whether there is a role for hormonal, lifestyle, cultural and/or socioeconomic factors in sex-specific risk factor control is unclear.

### Sex Hormones and Diabetes

Several studies have suggested that endogenous sex hormones play a role in type 2 diabetes development [47–50]. In particular, significant sex differences were observed for the association between endogenous testosterone and risk of type 2 diabetes [47]. Ding et al. showed that in postmenopausal women, testosterone levels are related to increased risk of developing type 2 diabetes [48]. The authors hypothesized that this potentially causal relationship may be due to testosterone-dependent impairment of insulin-mediated glucose uptake and increase in lipogenesis [48]. Furthermore, Kalyani et al. reported that in postmenopausal women, after adjustment for body mass and insulin resistance, the association between higher bioavailable testosterone and type 2 diabetes incidence was no longer present, suggesting that adiposity and insulin resistance mediate this association [50]. Androgen excess in women likely impairs insulin action, e.g. women with polycystic ovarian syndrome (PCOS) are predisposed to type 2 diabetes [51].

Recently, positive correlations have been reported between both testosterone and oestrogen levels and incidence of type 2 diabetes in postmenopausal women [52]. However, other published data question the existence of a causative link between oestrogen and diabetes [48, 50]. Moreover, it has been hypothesized that the increased cardiovascular risk in postmenopausal women could be partially caused by the loss of protection conferred by endogenous oestrogen [53]. However, a causal relationship between sex hormones and diabetes-associated cardiovascular risk has not been demonstrated.

## Future Perspectives

The often dramatically higher cardiovascular risk that is observed in women with diabetes highlights the need for more intensive treatment and optimized management of cardiovascular risk factors in diabetic and pre-diabetic women [23, 35]. The onset of type 2 diabetes is believed to occur years before its clinical diagnosis [54]. This suggests that individuals with pre-diabetes are being exposed to cardiovascular risk factors associated with diabetes even before the disease becomes manifest. Screening for pre-diabetes, combined with more stringent follow-up of women at high risk for diabetes, such as those with a history of gestational diabetes, polycystic ovary syndrome, or postmenopausal women, could have a substantial impact on the prevention of cardiovascular disease.

The reasons for the increased cardiovascular risk in women with diabetes are unclear, and this disparity is unlikely to be explained by gender differences in pharmacotherapy [24, 31]. As discussed before, sex differences in cardiovascular risk factors have been reported, but are unlikely to be the only cause of the excess cardiovascular risk in diabetic women [31].

Further sex-specific analyses of large-scale epidemiological studies that include individuals with and without diabetes are needed to elucidate the causes of the increased cardiovascular complications observed in women with diabetes. The ongoing Maastricht Study, which aims to include *n* = 10,000 men and women with a large proportion of individuals with diabetes in whom extensive phenotyping will be performed using state-of-the-art techniques is designed to address this need. Collection of detailed information on conventional and emerging cardiovascular risk factors, socioeconomic and lifestyle factors, preclinical cardiac and vascular abnormalities and cardiovascular outcomes (among other comorbidities) in both men and women is expected to contribute importantly to the understanding of sex-specific mechanisms underlying diabetic complications. Begun in 2010, the Maastricht Study is one of the largest prospective cohort studies of its kind [55]. Enhanced elucidation of sex-specific risk factors will provide opportunities for the development of novel prevention and treatment strategies. Because gender disparities in risk factor control exist even when women and men are treated similarly, better understanding of the sex-specific effects of risk factor-modifying interventions such as pharmacotherapy or lifestyle changes is much needed. Future trials of risk factor management in individuals with diabetes should take into account sex differences in order to develop more effective prevention and treatment strategies aimed at reducing cardiovascular events in both women and men.

## Conclusion

Several studies have demonstrated that diabetes is more strongly associated with cardiovascular risk in women than it is in men. The underlying causes for the diabetes-related sex difference in cardiovascular risk are still unclear. Although roles of sex hormones and menopause have been hypothesized, sex differences in diabetes-associated cardiovascular risk factors are mainly believed to be responsible for this increased risk. Current evidence indicates that there is room for improvement of cardiovascular risk factor control in diabetic women. Moreover, increased screening for pre-diabetes and more stringent follow-up and risk factor management in female patients could strongly improve the prevention of cardiovascular disease. However, large-scale epidemiological studies are needed to further elucidate the factors that underlie the excess cardiovascular risk in diabetic women.

## References

[1] Danaei G, Finucane MM, Lu Y, Singh GM, Cowan MJ, Paciorek CJ (2011). National, regional, and global trends in fasting plasma glucose and diabetes prevalence since 1980: systematic analysis of health examination surveys and epidemiological studies with 370 country-years and 2 · 7 million participants. Lancet.

[2] International Diabetes Federation. IDF Diabetes Atlas update poster, 6th edn. Brussels Belgium: International Diabetes Federation, 2014. http://www.idf.org/diabetesatlas

[3] WHO Estimates for 2000–2012 http://www.who.int/healthinfo/global_burden_disease/estimates/en/index1.html

[4.•] International Diabetes Federation. IDF Diabetes Atlas, 6th edn. Brussels Belgium: International Diabetes Federation, 2013. http://www.idf.org/diabetesatlas **The International Diabetes Federation (IDF) is an umbrella organization of over 230 national diabetes associations in 170 countries and territories. The 6th Edition of the IDF Diabetes Atlas reports diabetes epidemiology using new studies that became available in 2013, and updated population estimates.**

[5] DIAMOND Project Group (2006). Incidence and trends of childhood type 1 diabetes worldwide 1990–1999. Diabet Med J Br Diabet Assoc.

[6] Soltesz G, Patterson CC, Dahlquist G, EURODIAB Study Group (2007). Worldwide childhood type 1 diabetes incidence—what can we learn from epidemiology?. Pediatr Diabetes.

[7] Pundziute-Lyckå A, Dahlquist G, Nyström L, Arnqvist H, Björk E, Blohmé G (2002). The incidence of Type I diabetes has not increased but shifted to a younger age at diagnosis in the 0–34 years group in Sweden 1983–1998. Diabetologia.

[8] Gale EA, Gillespie KM (2001). Diabetes and gender. Diabetologia.

[9] Weets I, De Leeuw IH, Du Caju MVL, Rooman R, Keymeulen B, Mathieu C (2002). The incidence of type 1 diabetes in the age group 0–39 years has not increased in Antwerp (Belgium) between 1989 and 2000: evidence for earlier disease manifestation. Diabetes Care.

[10] Kyvik KO, Nystrom L, Gorus F, Songini M, Oestman J, Castell C (2004). The epidemiology of Type 1 diabetes mellitus is not the same in young adults as in children. Diabetologia.

[11] Zimmet P, Alberti KG, Shaw J (2001). Global and societal implications of the diabetes epidemic. Nature.

[12] Roche MM, Wang PP (2013). Sex differences in all-cause and cardiovascular mortality, hospitalization for individuals with and without diabetes, and patients with diabetes diagnosed early and late. Diabetes Care.

[13] Roglic G, Unwin N (2010). Mortality attributable to diabetes: estimates for the year 2010. Diabetes Res Clin Pract.

[14] Morrish NJ, Wang SL, Stevens LK, Fuller JH, Keen H (2001). Mortality and causes of death in the WHO Multinational Study of Vascular Disease in Diabetes. Diabetologia.

[15] Taylor KS, Heneghan CJ, Farmer AJ, Fuller AM, Adler AI, Aronson JK (2013). All-cause and cardiovascular mortality in middle-aged people with type 2 diabetes compared with people without diabetes in a large U.K. primary care database. Diabetes Care.

[16] Van Dieren S, Beulens JWJ, van der Schouw YT, Grobbee DE, Neal B (2010). The global burden of diabetes and its complications: an emerging pandemic. Eur J Cardiovasc Prev Rehabil Off J Eur Soc Cardiol Work Groups Epidemiol Prev Card Rehabil Exerc Physiol.

[17] Booth GL, Kapral MK, Fung K, Tu JV (2006). Relation between age and cardiovascular disease in men and women with diabetes compared with non-diabetic people: a population-based retrospective cohort study. Lancet.

[18.••] Kappert K, Böhm M, Schmieder R, Schumacher H, Teo K, Yusuf S (2012). Impact of sex on cardiovascular outcome in patients at high cardiovascular risk: analysis of the Telmisartan Randomized Assessment Study in ACE-Intolerant Subjects With Cardiovascular Disease (TRANSCEND) and the Ongoing Telmisartan Alone and in Combination With Ramipril Global End Point Trial (ONTARGET). Circulation.

[19] Anand SS, Islam S, Rosengren A, Franzosi MG, Steyn K, Yusufali AH (2008). Risk factors for myocardial infarction in women and men: insights from the INTERHEART study. Eur Heart J.

[20.••] Huxley RR, Peters SAE, Mishra GD, Woodward M. Risk of all-cause mortality and vascular events in women versus men with type 1 diabetes: a systematic review and meta-analysis. Lancet Diabetes Endocrinol 2015. **This is a meta-analysis of data from 26 studies, including 214,114 individuals and 15,273 events (14,682 deaths). This study showed that the excess fatal cardiovascular risk in women with type 1 diabetes is twice as high compared with men with type 1 diabetes** 10.1016/S2213-8587(14)70248-7 25660575

[21] Lee WL, Cheung AM, Cape D, Zinman B (2000). Impact of diabetes on coronary artery disease in women and men: a meta-analysis of prospective studies. Diabetes Care.

[22] Kanaya AM, Grady D, Barrett-Connor E (2002). Explaining the sex difference in coronary heart disease mortality among patients with type 2 diabetes mellitus: a meta-analysis. Arch Intern Med.

[23] Huxley R, Barzi F, Woodward M (2006). Excess risk of fatal coronary heart disease associated with diabetes in men and women: meta-analysis of 37 prospective cohort studies. BMJ.

[24.••] Peters SAE, Huxley RR, Woodward M (2014). Diabetes as risk factor for incident coronary heart disease in women compared with men: a systematic review and meta-analysis of 64 cohorts including 858,507 individuals and 28,203 coronary events. Diabetologia.

[25] Banerjee C, Moon YP, Paik MC, Rundek T, Mora-McLaughlin C, Vieira JR (2012). Duration of diabetes and risk of ischemic stroke: the Northern Manhattan Study. Stroke J Cereb Circ.

[26] Hyvärinen M, Tuomilehto J, Laatikainen T, Söderberg S, Eliasson M, Nilsson P (2009). The impact of diabetes on coronary heart disease differs from that on ischaemic stroke with regard to the gender. Cardiovasc Diabetol.

[27] Doi Y, Ninomiya T, Hata J, Fukuhara M, Yonemoto K, Iwase M (2010). Impact of glucose tolerance status on development of ischemic stroke and coronary heart disease in a general Japanese population: the Hisayama study. Stroke J Cereb Circ.

[28] Tanizaki Y, Kiyohara Y, Kato I, Iwamoto H, Nakayama K, Shinohara N (2000). Incidence and risk factors for subtypes of cerebral infarction in a general population: the Hisayama study. Stroke J Cereb Circ.

[29] Hu G, Jousilahti P, Qiao Q, Katoh S, Tuomilehto J (2005). Sex differences in cardiovascular and total mortality among diabetic and non-diabetic individuals with or without history of myocardial infarction. Diabetologia.

[30] Cui R, Iso H, Yamagishi K, Saito I, Kokubo Y, Inoue M (2011). Diabetes mellitus and risk of stroke and its subtypes among Japanese: the Japan public health center study. Stroke J Cereb Circ.

[31] Peters SAE, Huxley RR, Woodward M (2014). Diabetes as a risk factor for stroke in women compared with men: a systematic review and meta-analysis of 64 cohorts, including 775,385 individuals and 12,539 strokes. Lancet.

[32] Rautio A, Eliasson M, Stegmayr B (2008). Favorable trends in the incidence and outcome in stroke in nondiabetic and diabetic subjects: findings from the Northern Sweden MONICA Stroke Registry in 1985 to 2003. Stroke J Cereb Circ.

[33] Winell K, Pääkkönen R, Pietilä A, Reunanen A, Niemi M, Salomaa V (2011). Prognosis of ischaemic stroke is improving similarly in patients with type 2 diabetes as in nondiabetic patients in Finland. Int J Stroke Off J Int Stroke Soc.

[34] Venketasubramanian N, Röther J, Bhatt DL, Pasquet B, Mas J-L, Alberts MJ (2011). Two-year vascular event rates in patients with symptomatic cerebrovascular disease: the REACH registry. Cerebrovasc Dis Basel Switz.

[35] Eriksson M, Carlberg B, Eliasson M (2012). The disparity in long-term survival after a first stroke in patients with and without diabetes persists: the Northern Sweden MONICA study. Cerebrovasc Dis Basel Switz.

[36] Mansfield MW, Heywood DM, Grant PJ (1996). Sex differences in coagulation and fibrinolysis in white subjects with non-insulin-dependent diabetes mellitus. Arterioscler Thromb Vasc Biol.

[37] Howard BV, Cowan LD, Go O, Welty TK, Robbins DC, Lee ET (1998). Adverse effects of diabetes on multiple cardiovascular disease risk factors in women. The Strong Heart Study Diabetes Care.

[38] Juutilainen A, Kortelainen S, Lehto S, Rönnemaa T, Pyörälä K, Laakso M (2004). Gender difference in the impact of type 2 diabetes on coronary heart disease risk. Diabetes Care.

[39] Wannamethee SG, Papacosta O, Lawlor DA, Whincup PH, Lowe GD, Ebrahim S (2012). Do women exhibit greater differences in established and novel risk factors between diabetes and non-diabetes than men? The British Regional Heart Study and British Women’s Heart Health Study. Diabetologia.

[40] Steinberg HO, Paradisi G, Cronin J, Crowde K, Hempfling A, Hook G (2000). Type II diabetes abrogates sex differences in endothelial function in premenopausal women. Circulation.

[41] Han TS, Sattar N, Williams K, Gonzalez-Villalpando C, Lean MEJ, Haffner SM (2002). Prospective study of C-reactive protein in relation to the development of diabetes and metabolic syndrome in the Mexico City Diabetes Study. Diabetes Care.

[42] Kautzky-Willer A, Kamyar MR, Gerhat D, Handisurya A, Stemer G, Hudson S (2010). Sex-specific differences in metabolic control, cardiovascular risk, and interventions in patients with type 2 diabetes mellitus. Gend Med.

[43] Gouni-Berthold I, Berthold HK, Mantzoros CS, Böhm M, Krone W (2008). Sex disparities in the treatment and control of cardiovascular risk factors in type 2 diabetes. Diabetes Care.

[44] Franch-Nadal J, Mata-Cases M, Vinagre I, Patitucci F, Hermosilla E, Casellas A (2014). Differences in the cardiometabolic control in type 2 diabetes according to gender and the presence of cardiovascular disease: results from the eControl study. Int J Endocrinol.

[45] Franzini L, Ardigò D, Cavalot F, Miccoli R, Rivellese AA, Trovati M (2013). Women show worse control of type 2 diabetes and cardiovascular disease risk factors than men: results from the MIND.IT Study Group of the Italian Society of Diabetology. Nutr Metab Cardiovasc Dis NMCD.

[46.•] Penno G, Solini A, Bonora E, Fondelli C, Orsi E, Zerbini G (2013). Gender differences in cardiovascular disease risk factors, treatments and complications in patients with type 2 diabetes: the RIACE Italian multicentre study. J Intern Med.

[47] Ding EL, Song Y, Malik VS, Liu S (2006). Sex differences of endogenous sex hormones and risk of type 2 diabetes: a systematic review and meta-analysis. JAMA.

[48] Ding EL, Song Y, Manson JE, Rifai N, Buring JE, Liu S (2007). Plasma sex steroid hormones and risk of developing type 2 diabetes in women: a prospective study. Diabetologia.

[49] Ding EL, Song Y, Manson JE, Hunter DJ, Lee CC, Rifai N (2009). Sex hormone-binding globulin and risk of type 2 diabetes in women and men. N Engl J Med.

[50] Kalyani RR, Franco M, Dobs AS, Ouyang P, Vaidya D, Bertoni A (2009). The association of endogenous sex hormones, adiposity, and insulin resistance with incident diabetes in postmenopausal women. J Clin Endocrinol Metab.

[51] Jayasena CN, Franks S (2014). The management of patients with polycystic ovary syndrome. Nat Rev Endocrinol.

[52] Gubbels Bupp MR. Sex, the aging immune system, and chronic disease. Cell Immunol 2015; 10.1016/j.cellimm.2015.02.002 25700766

[53] Boukhris M, Tomasello SD, Marzà F, Bregante S, Pluchinotta FR, Galassi AR (2014). Coronary heart disease in postmenopausal women with type II diabetes mellitus and the impact of estrogen replacement therapy: a narrative review. Int J Endocrinol.

[54] Hu FB, Stampfer MJ, Haffner SM, Solomon CG, Willett WC, Manson JE (2002). Elevated risk of cardiovascular disease prior to clinical diagnosis of type 2 diabetes. Diabetes Care.

[55] Schram MT, Sep SJS, van der Kallen CJ, Dagnelie PC, Koster A, Schaper N (2014). The Maastricht Study: an extensive phenotyping study on determinants of type 2 diabetes, its complications and its comorbidities. Eur J Epidemiol.

